# The Needs Fulfillment of Medical Specialist in General Hospital Type C in Remote Areas

**Published:** 2019-07

**Authors:** Indriya PURNAMASARI, Dumilah AYUNINGTYAS, Ni Nyoman Dwi SUTRISNAWATI

**Affiliations:** Department of Health Administration and Policy, Faculty of Public Health, Universitas Indonesia, Depok, West Java, Indonesia

**Keywords:** Medical specialist, Low retention, Fulfillment efforts, Type c district hospital, Remote areas

## Abstract

**Background:**

Indonesia as an archipelagic country, makes the efforts to fulfill the needs of medical specialists in remote areas are not easy tasks. This study aimed to explore the availability and the needs fulfillment efforts of medical specialist in remote areas; and to know whether the availability of medical specialists have met the hospital needs, and how the authorities efforts to fulfill them, also the factors that affect the effort.

**Methods:**

This qualitative research was conducted with case study approach in Haji Abdoel Madjid Batoe District Hospital (RSUD HAMBA) Batanghari Jambi and Malingping District Hospital (RSUD Malingping) in Lebak Banten. In-depth interviews were conducted with various stakeholders.

**Results:**

The availability of medical specialist in both hospitals in number and type are inadequate. This fact indicates low retention and motivation of medical specialists to work in remote area. The major factor in both hospitals is inadequate salaries, difficult access, lack of medical equipment, also the difficulty to develop a career. However, these factors in both area ultimately boil down to the lack of government policies that support medical specialist fulfillment.

**Conclusion:**

The important factors that affect the lackness of effort in fulfilling medical specialists’ placement in remote areas are inadequate of infrastructures, competence and career development contraints, and the coordination between national and regional policies. The commitment of central and regional government to improve the infrastructures, competence and career developments, and regulations with respect to medical specialists in remote areas.

## Introduction

Limited human resources (HR) is one of the main hurdles in Indonesian health sector. The medical specialist nowadays is one of the health professions unequally distributed across the country. Medical specialist is still very limited, both in terms of quantity and types. Medical specialists preferred to work in big cities rather than in remote areas, whereas most of Indonesian people settle in remote areas. This condition is also reflected in the result of a study conducted by WHO-South East Asia Region (WHO SEARO) in Human Resources for Health-Country Profile Template: Indonesia 2010 (1–3).

In the regional autonomy era, the responsibility of health sectors, including the fulfillment of specialist physicians is the responsibility of the regional government (4). On the other hand, the Regional Government Law also emphasized the obligation of the National Government to fulfill the availability of specialists across the country, as it is crucial and still relatively rare.

In Indonesia, a study about factors that affects the placement of specialist physicians (bond) showed that the majority of medical specialists has low commitment and motivation to serve in remote areas. The reasons may come from the inadequate regional facilities to work and develop a career, and also the rewards obtain are below expectation (5).

The specialist who work in remote areas usually placed in district hospital (RSUD). Based on the decree from the Minister of Health No.56, 2014, about the Classification and Hospital Licensing, district hospitals are categorized as type C, especially if it is located in rural areas (6). If we referred to the decree, State General Hospital type C should have at least two specialists for every major specialized medical service; one specialist for every minor specialized medical support; and one specialist for every specialized dentistry.

As the number of Indonesia’s population continues to grow, there is also a need to increase the ratio of medical specialists from the 8.14 for 100,000 populations in 2010, to 12 for 100,000 population in 2014 (7).

Sumatra and Java are the two most populated islands in Indonesia. In Sumatra, medical specialists are concentrated in North Sumatra 8.62 for 100,000 population and West Sumatra of 8.25 for 100,000 population. Jambi, Bengkulu, and Lampung have the lowest ratio in Sumatra, with an average ratio below 4.00 for 100000 population (8). Meanwhile, Java as the most populous island in Indonesia, Jakarta topped with specialists with ratio 46.95 for 100,000 population. On the other hand, the lowest ratio in Java is Banten, which is 5.69 medical specialists for every 100,000 population (8).

The discrepancy of the medical specialist in the two Islands motivates the researcher to examine the fulfillment of medical specialists in those areas. Researchers then select two provinces, Jambi as the representative of Sumatra and Banten as the representative of Java, since both are the provinces with the lowest ratio of medical specialists than other provinces on the same island. The researcher conducts a preliminary study on two hospitals in two provinces namely Haji Abdoel Madjid Batoe District Hospital, Batanghari Jambi and Malingping District Hospital Banten to determine the availability of specialists. Based on the preliminary study, there are 10 specialized physicians in, and the ratio of specialists for 100,000 population in Batanghari is 3.87. Meanwhile, there are 9 specialized physicians in Malingping District Hospital and the ratio is less than 1.5.

There were efforts undertaken to meet the needs of medical specialists in both hospitals, but those efforts have not run optimally because there are other factors that affect the implementation. This creates an interest to conduct further investigation on the efforts to address the needs of medical specialist in District Hospital Type C in remote areas.

The main questions for this research are:

1. How are the availability and retention of medical specialists in HAMBA District Hospital Batanghari, Jambi Province and Malingping District Hospital, Banten Province as the C type hospitals in remote areas?
2. What efforts made to meet the needs of medical specialists in both areas as well as the various factors that influence?

The objectives of this study were to determine the availability of medical specialists in both remote areas based on the standard ratio and standard of requirements as described above, as well as to explore the various factors that affect the fulfillment of medical specialists there.

## Methods

This is qualitative method research, in a case study design. The data were obtained by literature studies, observation, and in-depth interviews with all related parties.

- Literature Study
  Data were also collected through literature studies, through various textbooks, articles in national and international indexed scientific journals, in addition to browsing through the internet electronic literature.
- In-depth Interviews
  The data through In-depth Interviews were collected in both national and regional levels. In national level, researchers have conducted in-depth interview with among others :
  1. Government officials associated with the Indonesian Ministry of Health
  2. The General Secretary of the Executive Board of the Indonesian Medical specialists Association (IDI)
  3. The Chairman of PB, Indonesian Hospital Association (PB PERSI)
  4. The Chairman of the Association of Regional Hospital (ARSADA).
  Informants at the regional level consist of :
  1. The relevant officials in the Provincial Government of Banten and Jambi
  2. The relevant officials in Batanghari regency
  3. Director and Head of Health Services of HAMBA District Hospital
  4. The Director of Malingping District Hospital and its subdivision
  5. The medical specialist in both hospitals
  6. Leaders of the regional community
  7. Representatives of the patients in both hospitals.
  For the interviews questions were slightly different depends on the position and authority of the informants. The interview questions for the Government official and Management of hospitals are:
  1. The availability of medical specialist at the Hospital (4 questions)
  2. Financial Remuneration (4 questions)
  3. Location and Geographical Condition (4 questions)
  4. Medical Equipment and Facilities in a Hospital (4 questions)
  5. Competence and Career Development (2 questions)
  6. Guidance and Supervision (4 questions)
  7. Regional Infrastructure (5 questions)
    All the results of in-depth interviews carefully recorded by the researchers and transcribed in the form of interview transcripts.
- Observation
  Observation in this research was made by observing hospital situation, the availability of medical specialist, and the impact of a specialist shortage on hospital’s services. The researcher also collected all relevant secondary data available.

This research was conducted in the related institutions focused on Jakarta, Jambi, Muara Bulian, Serang, and Malingping-Lebak. All data collected were analyzed in the process of researching so obtained results and conclusions.

## Results

### The Availability of Medical Specialist

In a glance, the availability of medical specialist in Type C District Hospitals in remote areas in the targeted research settings still below par based on the Minister of Health Regulation No. 56, Year 2014 on Classification and Hospital Licensing. Based on the in-depth interviews from the entire informant and research observation, the researcher obtained data that indicates both HAMBA District Hospital and Malingping District Hospital fail to adhere with the standard of a Type C District Hospital.

The distribution of types and numbers of medical specialist in both hospitals are not equal (Table 1).

### Financial Remuneration

Financial Remuneration provided by the local government of Batanghari is in form of profession scarcity incentives (HONDA) for the medical specialists with the status of civil servants, where the mechanism refers to the Regent Decree.

Currently, the amount of HONDA is 10 million rupiahs outside of monthly salary and specialized medical service given based on the number of patients. As of 2016, the number has been increased to 15 million rupiahs. Other than medical specialist employed by the government for guest specialist there is compensation fee of one million rupiahs. The funds are allocated from the Batanghari annual budget.

**Table 1:** Medical Specialist Data in Batanghari and Malingping District Hospital 2015

***Type C District Hospital***	***Main Specialist***								***Supporting Specialist***						***Other***	***Total***
	***Internal Medicine Physician***		***Pediatrician***		***Surgeon***		***Obstetrician Gynecologist***		***Anesthesiologist***		***Pathologist***		***Radiation Oncologist***			
	***NA***	***MR***	***NA***	***MR***	***NA***	***MR***	***NA***	***MR***	***NA***	***MR***	***NA***	***MR***	***NA***	***MR***		
*Haji Abdoel Madjid Batoe Kabupaten Batanghari*	*1*	*2*	*1*	*2*	*3*	*2*	*3*	*2*	*1*	*1*	*-*	*1*	*-*	*1*	*1 Eye*	*10*
*Malingping, Provinsi Banten*	*2*	*2*	*1*	*2*	*2*	*2*	*-*	*2*	*2*	*1*	*1*	*1*	*-*	*1*	*1 Ear-Throat*	*8*

Malingping District hospital as Banten Provincial Government owned institution, refer to Governor Decree and Price Unit Standard for all of the regulation in the hospital including financial incentives. Medical specialist employed by the government, they will receive 30 million rupiahs outside of salary and services. While contract-based employees receive one million rupiahs for every session.

“..during this time we get allowance 10 million per month. Certainly, it is not enough for a medical specialist in remote areas,.”

The condition in Malingping District Hospital is different. The contract medical specialist states that they have enough income earned from Malingping District Hospital. Instead, the senior ones who have a status of civil servants (PNS) actually disagree with the opinion. The overall income received is not currently in line with expectations, especially for the medical services or services whose counting mechanism and distribution are not done properly.

“.....we appreciate the government’s commitment to provide financial compensation. Although nominal amount looks great, but in fact it is still inadequate if we look at the working conditions in Malingping.”

There is a big difference in the salary amount for the PNS specialists in HAMBA District Hospital dan Malingping District Hospital. They think that big allowances are not enough and do not make them satisfied considering the places, and the limited conditions. The financial compensation is one important factor in addressing their needs there.

### Location and Geographical Condition

Malingping district is located, in the southernmost part of Banten Province. Being separated 300 km from Jakarta, the capital city of Indonesia, the time needed to reach Malingping from Jakarta ranges from 8–10 h.

The condition of the region is very quiet, very remote and far from other places. The participants stated that those factors might affect the availability of specialized physicians in Malingping.

Meanwhile, Batanghari District is located in a strategic location, located in the east traffic lane of Sumatra Island, and the closest to the capital of Jambi Province.

### Medical Equipment and Facilities in a Hospital

The facilities, medical and support equipment in HAMBA District Hospital are more sufficient and more complete than those in Malingping District Hospital.

The conditions in Malingping District Hospital was quite apprehensive. The hospital is classified as type C hospital considering its status as a property of the Provincial Government, but the various aspects are still not in the standard of type C.

### Competence and Career Development

HAMBA District Hospital and Malingping District Hospital management have special fund to give an opportunity to develop a career and competences for medical specialist. HAMBA District Hospital management provides the budget for education and training for each medical specialist, at least once a year.

Similarly, Malingping District Hospital provides funding of human resource development in the form of training or seminar/workshop package; however, there were no medical specialists have them, because of complicated procedures and lack of information about the availability of these funds.

### Guidance and Supervision

There were no guidance, supervision or monitoring and evaluation (M & E) to specialists for the technical and staff administration from the national and regional government, in both hospitals.

### Regional Infrastructure

The infrastructure condition in Batanghari Regency of Jambi Province is far better than in Malingping District of Banten Province. Muarabulian as the capital of Batanghari district has adequate regional infrastructure and public facilities for a district capital. Public transports in or between cities have already been available in the city.

This condition is different in Lebak District, where Malingping District Hospital is. The regional development of Malingping is still not optimal with many infrastructure and public facilities limitations. The poor road access to Malingping is one main reason for the specialists do not want to work to Malingping.

### Government Policy

#### National Government Policy

Most respondents in Jambi, especially in Batanghari and HAMBA District Hospital stated that there were no regulations or government policies regulating explicitly and in details about regional government obligation to fulfill the right specialists devoted to the hospitals. In Malingping District Hospital, the national government does not have a regulation on compensation standards for medical specialist who work in hospitals, especially in remote areas.

#### b. Regional or Local Government Policy

Jambi Province and Banten Province, where the regional government and legislation has issued their own policy in the effort for fulfilling the needs for strategic medical personnel in regional district such as medical specialist, still refers to the national government policy. However, this policy, which accommodates the needs of medical specialist, is still inadequate.

Apart from that, the difficulty to recruit and attract medical specialist to serve in Batanghari hospitals creates more hurdles to strive towards standardized health services. Up until now, regional government has not been able to find effective measures to recruit medical specialist, even though collaboration with numerous education centers continually improved. Regional government is continually improving its efforts to recruit medical specialist through collaboration with universities.

## Discussion

The novelty or exclusiveness of this research is provide an overview or mapping of the availability and the efforts to meet the needs of medical specialists in Government Hospitals in remote areas, especially in the era of Decentralization or Regional Autonomy, greatly influenced by the political context (e.g. the distribution or preparation of human resource management between the central government and local governments with various political and social polemics). This topic has never been specifically studied, especially in the era of Regional Autonomy in Indonesia, which there is an attractive condition between the district and the province as well as the rules of the central government.

The holistic outlook of research findings conducted both in HAMBA and Malingping District Hospitals found that medical specialist availability in both hospitals are inadequate in both quantity and diversity; they are not coherent with the standard of C type General Hospital for medical specialist staff as stated in Ministry of Health Decree Number 56 year 2014 on Classification and Licensing (6).

Several factors which influence the motivation and the retention of medical specialist which affects the effort in accommodating the needs of medical specialist in both district hospitals covers easy access to reach the district, the availability of adequate financial incentives, the availability of non-financial incentives such as housing, transportation vehicle, as well as the opportunity to develop career and competence.

In Indonesia, finance has a strong correlation between the regional financial capabilities and the direction of regional governments in setting up the mechanisms of financial compensation for medical specialists. This is in line with the concept of the distribution balance of Health Human Resources as one of the determinants that affect the balance of the distribution of human resources for health, are relates to finance and plan (9).

Contradicted to those in Malingping District Hospital, the medical specialist in HAMBA District Hospital feel the satisfaction and adequacy of what they earn, and feel the comfort in working though they do not get big compensation, which indicates that the financial compensations in the form of salary/allowances are not always able to guarantee the motivation improvement of an employee if he did not get a sense of comfort in the environment in which they work. This fact suits to the Theory of Work Motivation, where there are several aspects influencing the motivation of one’s work; those are security in work, a fair and competitive salary, a pleasant working environment, reward for the high performance and fair treatment from the management.

The main factor of the retention of the human resource was career opportunities, in addition to components of the organization, awards, design of tasks and jobs and staff relations (10). There are five main determinants that affect the effort to fulfill the health human resources, including policy or regulation, adequate compensation, supervision, availability of support systems and career development (3).

Other factors are the availability of good infrastructure and public health system that covers medical equipment and supporting apparatus, safe and comfortable working condition, as well as good communication between medical specialist, director and managers, and finally regional government’s policy on medical specialist retention in the district.

As we know, guidance and supervision aspects also play important roles in determining the success of a program/activity/work. Referring to the regulations of Law No. 29 of 2004 about the Practice of Medicine and Decree No. 2052 of 2011 regarding the Practice License and Implementation of Medical Practice, the national government, regional government, and professional organizations give the tasks of carrying out the guidance and supervision on the implementation of practices medicine, including the practice of medical specialists (11). The aim was to ensure equity and quality improvement of health cares for all Indonesian.

Both hospitals have not implemented and conducted routine supervisions to specialists who practice in HAMBA District Hospital dan Malingping District Hospital as the functional tasks and authority yet. However, all informants agree that it is crucial matters.

On the other hand, the regional infrastructure and public facilities development still have not run well in Malingping because of geography location and conditions. Another important thing known is the lack of regional government’s coordination and communication between Lebak District Government and the Provincial Government. The influence of adequate infrastructure is to improve access and retention of human resources (12).

Policy is one of the most important factors in satisfying the needs and equal distribution of medical personnel, including medical specialist.

### Suggestion and Recommendation

To overcome the shortage of medical specialist, policy makers needs to create a list of medical specialist needed in certain areas by mapping the number and the kinds of medical specialist are not available in their district, therefore they can determine which kind of medical specialist needed in accordance with the number of population and the increase of population, the development of epidemiology of diseases, also the number and the kinds of medical specialist according to the standard of the hospital’s type. Hospital’s management needs to identify insufficient equipment and must make them available through the standard equipment and also create a list of suggestions and increase communication between medical specialists in order to create a comfortable working environment. Concerning political dynamics that frequently happens and influencing to the increased number of turn over health manpower in the regional districts, either in the regional leaders, officials and even operational staff, therefore the national government must strive in the effort for promoting understanding concerning philosophy, fundamental, and the objective of regional autonomy; to create equal distribution of development and welfare. National government in conjunction with the regional government must formulate and implementing a comprehensive and specifics policy that is concerning the medical specialist especially those that are placed in remote areas, covers the standard pattern of recruitment, placement, standard remuneration, rights and responsibility of medical specialist; by considering workload, risk factor, geography, condition of the region, and the condition of the district hospitals. These measures are taken to reduce the disparity of the remuneration given out to medical specialist. The involvement and contribution of regional government become pivotal in supporting national policy. Even so, the national government must carry out innovative approach to recruit and place medical specialist on a temporary basis for a certain time frame but sustainable, formulate the policies and actively participate in the development of public health system in the district hospital.

## Conclusion

Although not intended to generalize with any other areas, but the lesson learned from this study can be a universal learning and can be implemented to meet the needs of specialist physicians in the era of decentralization in remote areas with similar conditions; a lesson learned that in the level of bureaucratization, managing the fulfillment of medical specialist mainly in the remote areas is not an easy task.

## Ethical considerations

Ethical issues (Including plagiarism, informed consent, misconduct, data fabrication and/or falsification, double publication and/or submission, redundancy, etc.) have been completely observed by the authors.
